# Phosphorylation of murine SAMHD1 regulates its antiretroviral activity

**DOI:** 10.1186/s12977-015-0229-6

**Published:** 2015-12-15

**Authors:** Sabine Wittmann, Rayk Behrendt, Kristin Eissmann, Bianca Volkmann, Dominique Thomas, Thomas Ebert, Alexandra Cribier, Monsef Benkirane, Veit Hornung, Nerea Ferreirós Bouzas, Thomas Gramberg

**Affiliations:** Institute of Clinical and Molecular Virology, Friedrich-Alexander University Erlangen-Nürnberg, Schlossgarten 4, 91054 Erlangen, Germany; Institute for Immunology, Medical Faculty Carl Gustav Carus, University of Technology Dresden, Fetscherstrasse 74, 01307 Dresden, Germany; Pharmazentrum Frankfurt/ZAFES, Institute of Clinical Pharmacology, Goethe-University, Frankfurt Theodor Stern Kai 7, 60590 Frankfurt am Main, Germany; Institute of Molecular Medicine, University Hospital, University of Bonn, Bonn, Germany; Laboratoire de Virologie Moléculaire, Institut de Génétique Humaine, CNRS UPR1142, Montpellier, 34000 France

**Keywords:** HIV-1, MLV, SAMHD1 knockout mouse, SAMHD1 phosphorylation

## Abstract

**Background:**

Human SAMHD1 is a triphosphohydrolase that restricts the replication of retroviruses, retroelements and DNA viruses in noncycling cells. While modes of action have been extensively described for human SAMHD1, only little is known about the regulation of SAMHD1 in the mouse. Here, we characterize the antiviral activity of murine SAMHD1 with the help of knockout mice to shed light on the regulation and the mechanism of the SAMHD1 restriction and to validate the SAMHD1 knockout mouse model for the use in future infectivity studies.

**Results:**

We found that endogenous mouse SAMHD1 restricts not only HIV-1 but also MLV reporter virus infection at the level of reverse transcription in primary myeloid cells. Similar to the human protein, the antiviral activity of murine SAMHD1 is regulated through phosphorylation at threonine 603 and is limited to nondividing cells. Comparing the susceptibility to infection with intracellular dNTP levels and SAMHD1 phosphorylation in different cell types shows that both functions are important determinants of the antiviral activity of murine SAMHD1. In contrast, we found the proposed RNase activity of SAMHD1 to be less important and could not detect any effect of mouse or human SAMHD1 on the level of incoming viral RNA.

**Conclusion:**

Our findings show that SAMHD1 in the mouse blocks retroviral infection at the level of reverse transcription and is regulated through cell cycle-dependent phosphorylation. We show that the antiviral restriction mediated by murine SAMHD1 is mechanistically similar to what is known for the human protein, making the SAMHD1 knockout mouse model a valuable tool to characterize the influence of SAMHD1 on the replication of different viruses in vivo.

## Background

Genetic defects of proteins, which are involved the recognition or removal of intracellular nucleic acids have been shown to cause a dysregulated type I interferon (IFN) response that frequently results in autoimmunity [1]. In Aicardi-Goutières syndrome (AGS), which represents a rare monogenic variant of the prototypic autoimmune disease systemic lupus erythematosus, defects of either TREX1 [2], RNaseH2 (subunits A, B and C) [3], ADAR1 [4], MDA5 (Ifih1) [5], or SAMHD1 cause the cell to spontaneously produce large amounts of type I IFN [6]. The spontaneous cell-intrinsic activation of the innate antiviral immune response led to the speculation that these factors would also interfere with viral replication. Shortly after TREX1 has been shown to interfere with the replication of human immunodeficiency virus 1 (HIV-1) [7], SAMHD1 was identified as an antiretroviral restriction factor that is largely responsible for the inability of HIV-1 to infect cells of the myeloid lineage and resting T cells [8–11]. In addition to HIV-1, SAMHD1 has been shown to block the replication of various retroviruses, as well as DNA viruses like Herpes simplex virus, Vaccinia Virus, or Hepatitis B virus in myeloid cells [12–16]. Interestingly, only HIV-2 and simian immunodeficiency viruses (SIV) encode the accessory protein Vpx to counteract the restriction mediated by SAMHD1 in a species-specific manner [17–19]. Vpx has been shown to directly bind to SAMHD1 [8, 9]. At the same time it binds to a Cul4 E3 Ubiquitin ligase complex via the adapter molecule DCAF1 [20], which results in ubiquitination and proteasomal degradation of SAMHD1.

The mechanism by which SAMHD1 blocks viral infection, however, is controversially discussed. SAMHD1 acts as a dNTP triphosphohydrolase that cleaves dNTPs into nucleosides and inorganic triphosphates [21–23]. SAMHD1 is therefore believed to limit reverse transcription by depleting cellular dNTPs below the level sufficient for retroviral reverse transcription (RT). Alternatively, some reports describe nucleic acid binding and a nuclease activity of SAMHD1 [24–26]. In line with these findings, it has recently been shown that SAMHD1 inhibits HIV-1 infection through degradation of incoming genomic RNA [27]. However, since SAMHD1 has been described to be a nuclear protein, the mechanism how it degrades incoming viral RNA in the cytoplasm is not completely understood. It has been shown that phosphorylation of human SAMHD1 at threonine 592 (T592) by the cell cycle-dependent kinases 1 and 2 (CDK1 and CDK2) regulates the antiviral activity [28–30]. Initially, it has been suggested that the SAMHD1 phosphomimetic mutants T592D and T592E lose their antiviral activity but are still able to hydrolyze cellular dNTPs [28, 31, 32]. Based on these results an additional unknown mechanism of SAMHD1 restriction besides dNTP depletion has been proposed. However, recent work by three different groups showed independently that the phosphorylation of SAMHD1 at T592 downregulates the dNTP hydrolase activity of the protein, especially at low nucleotide concentrations [33–35]. These findings strongly suggest that the depletion of dNTPs by human SAMHD1 is the most likely mechanism of retroviral restriction.

To characterize the role of SAMHD1 in the onset of autoimmunity and viral restriction in vivo, we and others generated SAMHD1 knockout mice (SAMHD1 KO) [36, 37]. In the absence of viral infection, these mice show a transcriptional upregulation of interferon-stimulated genes (ISG) in various cell types indicative of spontaneous IFN production, a situation similar to human AGS patients. We detected increased dNTP levels in various cell types of knockout mice suggesting that endogenous murine SAMHD1 is an active phosphohydrolase in vivo. In retroviral infection assays we found that SAMHD1 blocks the infection of HIV reporter virus in vitro and in vivo. However, SAMHD1 expression did not affect the replication of Friend Murine Leukemia Virus (Friend MLV), a murine retrovirus, in the mouse model. Due to this discrepancy, we further characterized the mechanism of viral restriction and the regulation of the antiviral activity of murine SAMHD1 (muSAMHD1).

In this study, we found that endogenous muSAMHD1 blocks retroviral infection at the level of reverse transcription, while degradation of viral genomic RNA by muSAMHD1 seems to be less important. On the level of reverse transcription, muSAMHD1 not only restricted HIV reporter virus but also proved to be active against the murine retrovirus MLV. We also found that the antiviral activity of muSAMHD1 is negatively regulated through phosphorylation at threonine 603 (T603) in cycling cells. In noncycling primary murine cells and THP-1 cells expressing murine SAMHD1 isoforms, the inhibition of retroviral infection coincides with decreased dNTP levels. These findings suggest that the antiviral activity of muSAMHD1 is mediated by its dNTPase activity, which is regulated by the phosphorylation status of the protein.

## Results and discussion

### Mouse SAMHD1 blocks HIV reporter virus infectivity at the level of reverse transcription

**Fig. 1:**
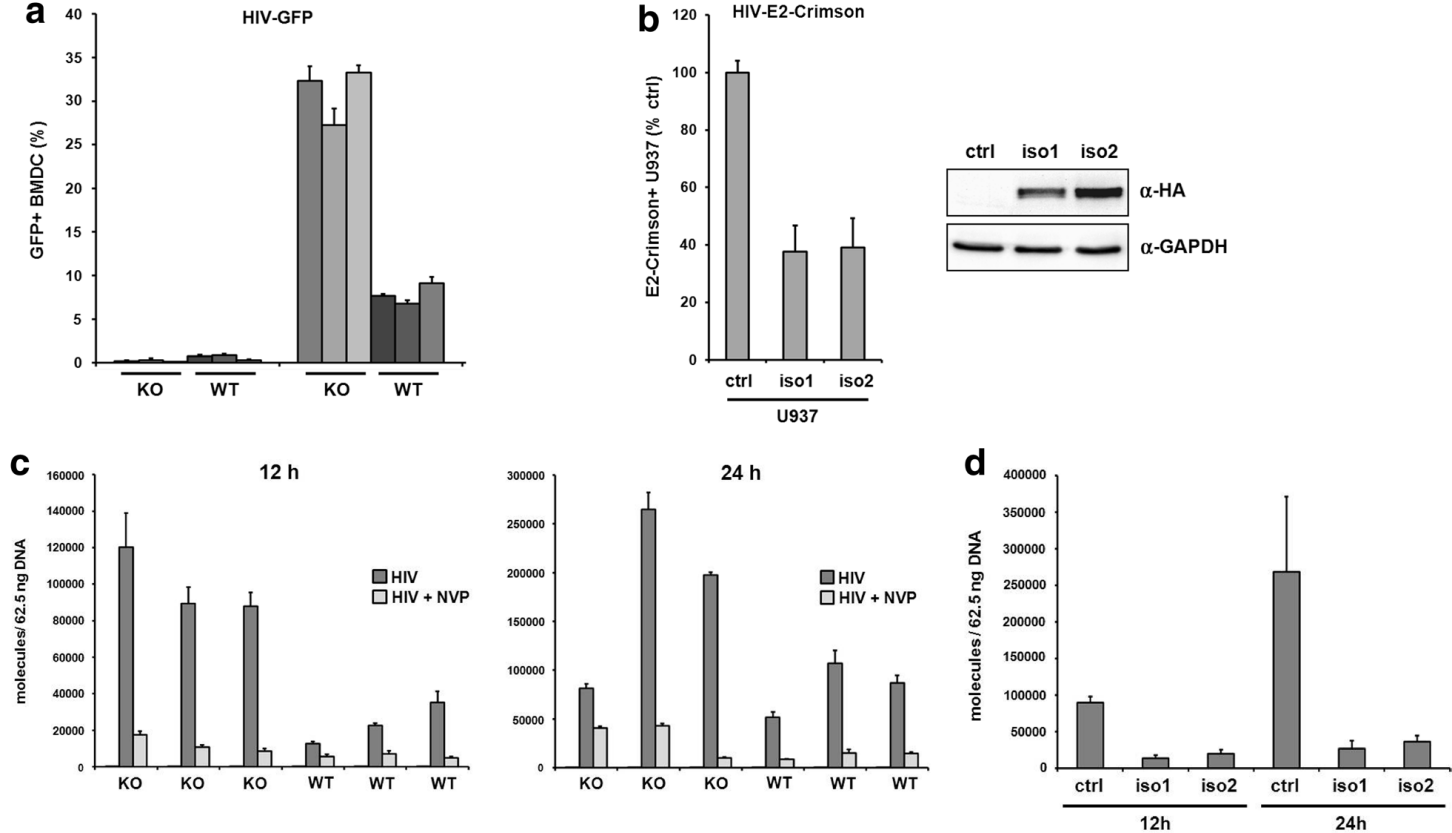
Mouse SAMHD1 inhibits lentiviral infection at the level of reverse transcription. **a** Bone marrow derived dendritic cells (BMDC) from wild-type (WT; n = 3) and SAMHD1 knockout mice (KO; n = 3) were either not infected (mock) or infected with VSV-G pseudotyped HIV-CMVGFP reporter virus (MOI of 1). Reporter virus infection was quantified 3 days later by flow cytometry. The percentage of infected cells (GFP+) is shown as average of triplicate infections with error bars indicating the standard deviation. One out of two independent experiments is shown. **b** PMA-treated U937 cells expressing murine isoform 1 (iso1), isoform 2 (iso2), or empty control vector were infected with VSV-G pseudotyped HIV-E2-Crimson reporter virus (MOI of 1). The percentage of infected cells has been determined 3 days later by flow cytometry. The columns represent the average of three independent experiments with error bars indicating the standard deviation. To control for SAMHD1 iso1 and iso2 expression, cell lysates were prepared and analyzed on an immunoblot probed with anti-HA MAb or anti-GAPDH antibody. **c** BMDC from WT (n = 3) or KO mice (n = 3) were incubated VSV-G/HIV-CMVGFP reporter virus at a MOI of 1. Cellular DNA was isolated 12 and 24 h postinfection and used as a template in qPCR to amplify HIV-CMVGFP reverse transcription products. Nevirapine (NVP) was added to the indicated wells 14 h prior to infection to control for contaminating plasmid DNA. The data are presented as the average of triplicates with *error bars* indicating the standard deviation. One out of three independent experiments is shown. **d** PMA-treated U937-control, U937-iso1, and U937-iso2 cells were incubated VSV-G/HIV-CMVGFP reporter virus at a MOI of 1. Total DNA was isolated from the cells at 12 and 24 h postinfection and used to amplify reverse transcription products by qPCR. The data are presented as the average of triplicates with *error bars* indicating the standard deviation. The results shown are representative of results obtained in at least three independent experiments

To determine the role of muSAMHD1 during retroviral infection, we first analyzed the antiviral activity of muSAMHD1 in primary bone marrow-derived dendritic cells (BMDC) and human myeloid U937 cells, which stably express the muSAMHD1 isoforms 1 and 2 individually. To assess the inhibitory role of endogenous SAMHD1, we infected SAMHD1 knockout (KO) and wild-type (WT) BMDC with a VSV-G pseudotyped HIV-CMVGFP reporter virus that expresses the GFP reporter gene under the control of a CMV promotor. Three days postinfection at a multiplicity of infection (MOI) of 1, we compared the reporter virus infectivity in WT and KO BMDC by flow cytometry. We found a nearly fivefold enhancement of HIV infectivity in BMDC from SAMHD1 KO mice compared to WT cells (Fig. 1a). Of note, preincubation of murine BMDCs with SIV virus-like particles (VLP) containing the HIV-2/SIV accessory protein Vpx did not counteract the antiviral activity of muSAMHD1 by inducing the degradation of endogenous SAMHD1 (data not shown). This is in line with previous sequence analysis and infection experiments showing that exogenously overexpressed muSAMHD1 is not degraded by Vpx [18, 19, 23], Next, we differentiated human U937 myeloid cells containing the mouse SAMHD1 isoforms 1 or 2 or an empty control vector into macrophage-like cells by incubating the cells with the phorbol ester PMA. The murine SAMHD1 splice variants differ in their C-terminal tail. Isoform 2 is lacking the potential phosphorylation site (T603), which has been shown to be important for the regulation of the antiviral function in the human protein. We found that both muSAMHD1 isoforms efficiently restrict HIV-1 reporter virus infection by almost threefold when overexpressed in our human myeloid cell line model (Fig. 1b).

The mechanism how human SAMHD1 inhibits retroviral infection is controversially discussed. Since human SAMHD1 displays a dNTP phosphohydrolase activity in vitro and in vivo, it has been suggested to inhibit reverse transcription by depleting the intracellular dNTP pool. To determine whether SAMHD1 in the mouse also affects reverse transcription (RT), we infected WT or SAMHD1 KO BMDC from different donor mice with HIV-1 reporter virus at a MOI of 1 and determined the number of reverse transcribed viral DNA molecules by quantitative PCR (Fig. 1c). After 12 and 24 h, we found enhanced levels of late reverse transcripts (late RT) in SAMHD1 KO BMDC compared to cells from WT mice (Fig. 1c). The effect was most pronounced at 12 h postinfection and resulted in a fivefold enhancement of viral RT products. Samples treated with the RT inhibitor nevirapine (NVP) were included in the infections. In the NVP control samples only a few molecules were detected, demonstrating the absence of contaminating plasmid DNA. Next, we determined whether both murine isoforms are able to inhibit viral RT. Therefore, we infected PMA-treated U937 cells that contain isoform 1, isoform 2, or a control plasmid with HIV-1 reporter virus and analyzed the viral DNA content by qPCR (Fig. 1d). The expression of both murine isoforms caused a significant reduction in the number of late RT transcripts 12 and 24 h postinfection indicating that both proteins block viral transduction at the level of reverse transcription. Together, these findings show that both isoforms of murine SAMHD1 are antiviral active and inhibit HIV reporter virus infection at or prior to the level of RT in a myeloid cell line and primary mouse BMDC.

### SAMHD1 blocks MLV reverse transcription in primary murine cells

**Fig. 2:**
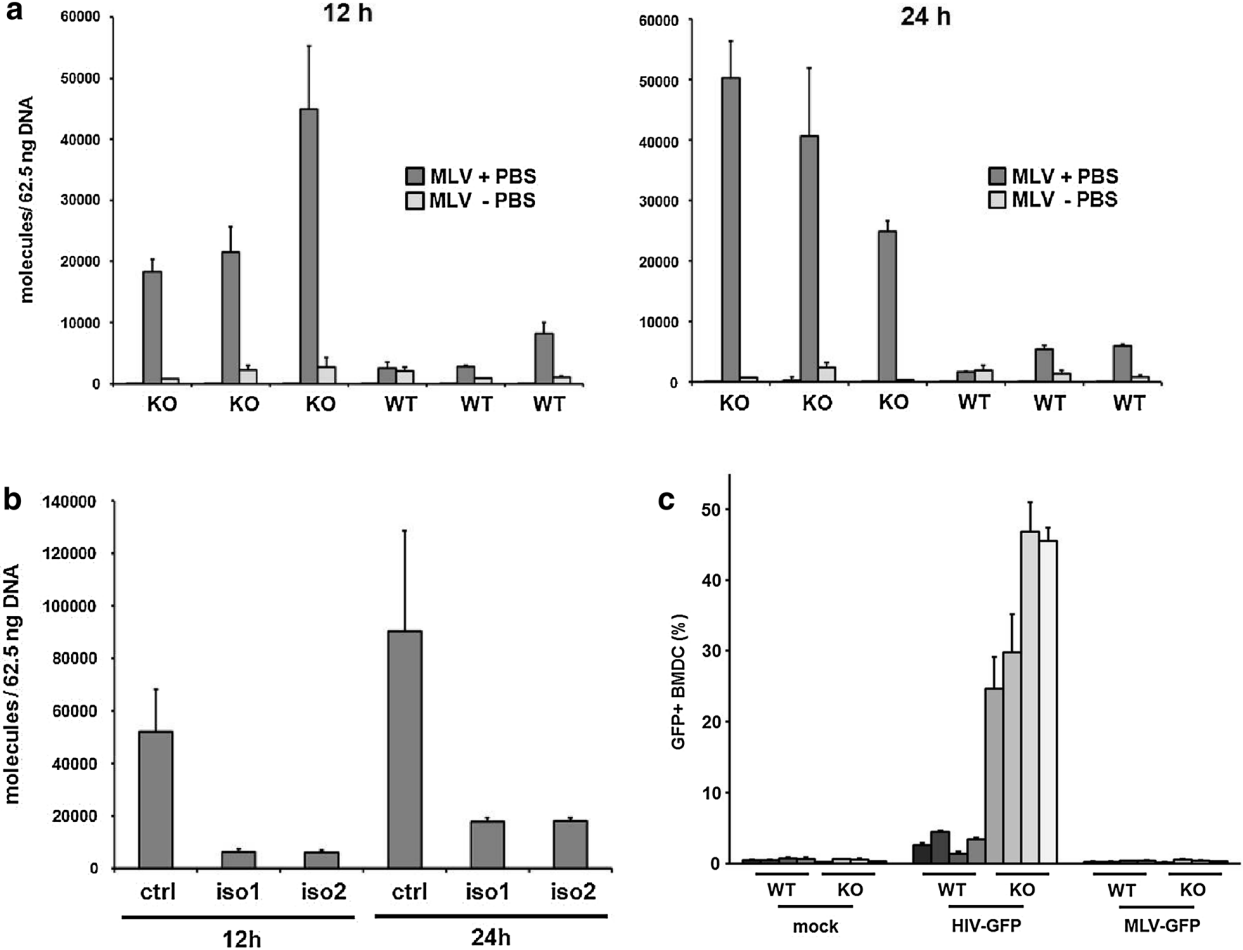
Murine SAMHD1 blocks reverse transcription of MLV in a myeloid cell line and primary BMDCs. **a** BMDC were incubated with VSV-G-pseudotyped MLV-GFP reporter virus with (MLV +PBS) or without (MLV −PBS) an intact Primer Binding Site (PBS) to control for contaminating plasmid DNA (MOI of 1). At 12 and 24 h postinfection, cellular DNA was harvested and MLV reverse transcripts were quantified by qPCR using oligonucleotides targeting the reporter gene sequence. The data are presented as the average of triplicates with error bars indicating the standard deviation. The data for one of three independent experiments is shown. **b** PMA-treated U937-control, U937-isoform 1, and U937-isoform 2 cells were incubated VSV-G/HIV-CMVGFP reporter virus at a MOI of 1. Cellular DNA was isolated at 12 and 24 h postinfection and used to amplify reverse transcription products by qPCR. The oligos used in qPCR were targeting the reporter gene sequence. The data are presented as the average of triplicates with error bars indicating the standard deviation. The results shown are representative of results obtained in three independent experiments. **c** BMDC from WT (n = 3) and SAMHD1 KO (n = 3) mice were either not infected (mock), infected with VSV-G/MLV-GFP, or infected with VSV-G/HIV-CMVGFP reporter virus at a MOI of 1. Viral infectivity was quantified 3 days postinfection by flow cytometry. The percentage of GFP + BMDC is shown as average of triplicate infections. One out of two independent experiments is shown

Previously, we compared the replication of Friend MLV in SAMHD1 KO and WT mice but could not detect any differences in Friend MLV replication capacity in vivo [36]. Since MLV only replicates efficiently in dividing cells, we speculated that SAMHD1 might be not active in Friend MLV target cells. However, we could not exclude that endogenous murine SAMHD1 might not be active against murine retroviruses. To determine whether endogenous mouse SAMHD1 is also active against a murine retrovirus, we infected BMDC from SAMHD1 KO and WT mice with a MLV-GFP reporter virus at a MOI of 1 and analyzed the accumulation of viral DNA over time by qPCR (Fig. 2a). For amplification of viral transcripts we used oligos targeting the GFP sequence of the reporter virus to avoid unspecific signals from integrated endogenous retroviral sequences. We detected a more than tenfold higher abundance of MLV late RT products in SAMHD1 KO BMDC compared to WT cells after 12 and 24 h. BMDC were also infected with a MLV reporter virus that lacks the primer binding site (MLV -PBS). Due to the missing interaction with cellular tRNAs, MLV -PBS cannot initiate reverse transcription. The MLV -PBS control samples contained only few molecules, demonstrating the absence of contaminating plasmid DNA. Next, we determined the influence of the two different murine splice variants on MLV RT and infected PMA-treated U937 cells expressing isoform 1, isoform 2 or control cells with MLV-GFP reporter virus at a MOI of 1 (Fig. 2b). After 12 and 24 h, we found strongly reduced numbers of MLV reverse transcripts in cells expressing SAMHD1 isoform 1 or 2, indicating that both murine SAMHD1 isoforms are able to inhibit MLV RT. Thus, muSAMHD1 is also blocking the infection of murine leukemia virus at the level of RT in primary cells. However, the relief of the block in SAMHD1 knockout cells does not lead to productive MLV infection of murine BMDC (Fig. 2c).

### Phosphorylation of murine SAMHD1 at threonine 603 correlates with its antiviral activity

**Fig. 3:**
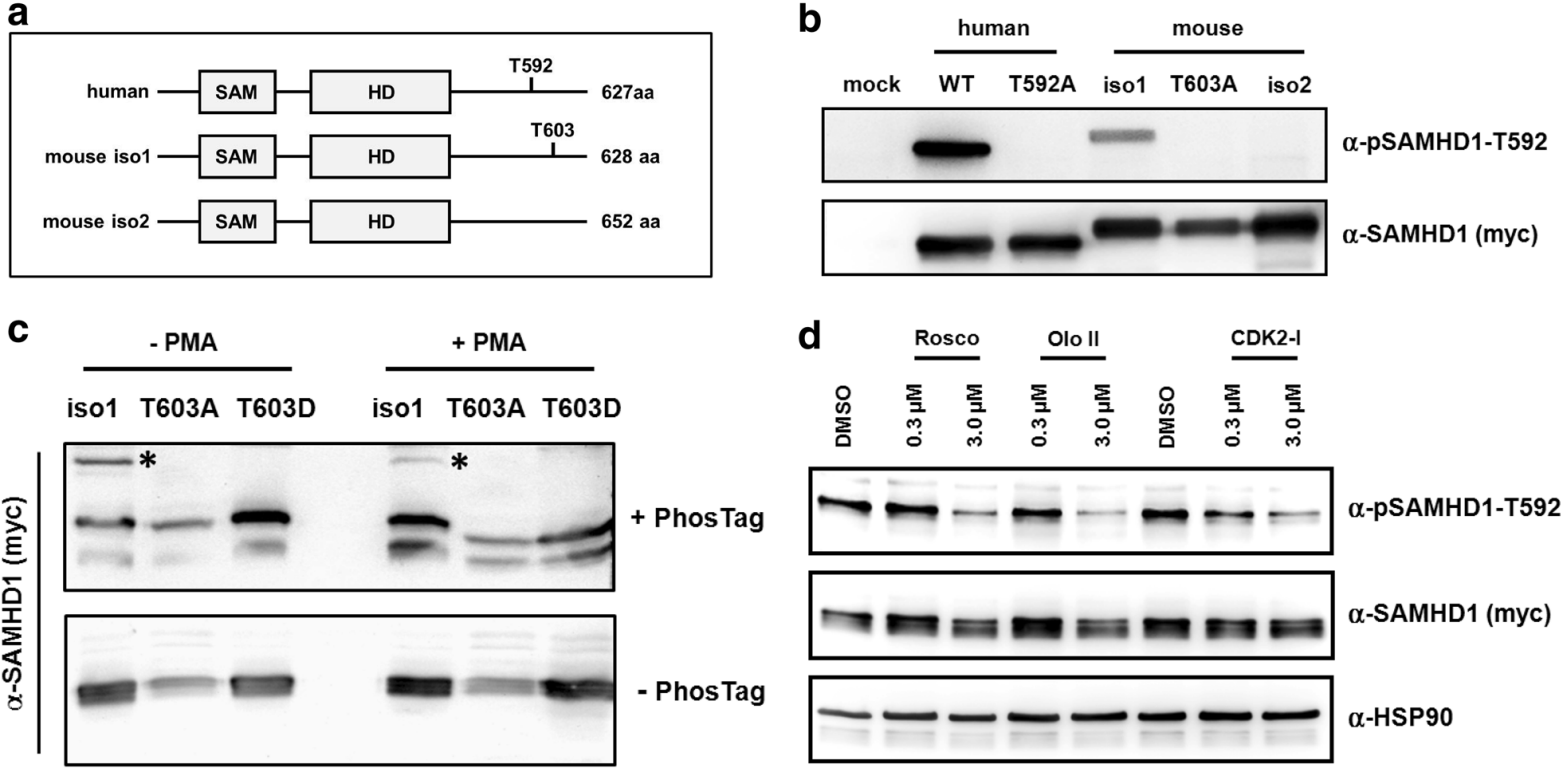
Murine SAMHD1 is phosphorylated at threonine 603 in cycling cells. **a** Schematic overview of the different SAMHD1 constructs used in the study. Human SAMHD1 and both murine isoforms contain the SAM domain and the enzymatic active HD Domain. Phosphorylation of threonine 592 in human SAMHD1 regulates its antiviral activity. The corresponding threonine is at position 603 in murine isoform 1 (iso1), but is absent in the isoform 2 (iso2) sequence due to differential splicing. **b** 293T cells were co-transfected with expression plasmids for 3′myc-tagged human SAMHD1, human SAMHD1-mutant T592A (T592A), mouse iso1, iso1-mutant T603A (T603A), or murine iso2. After 2 days, cell lysates were prepared and analyzed on an immunoblot probed with anti-myc MAb, or anti-pSAMHD1-T592 antibody. **c** THP-1 huSAMHD1 KO cells stably expressing 3′myc-tagged murine SAMHD1 isoform 1 (iso1), iso1-T603A, or iso1-T603D were incubated with 50 nM PMA (+PMA) or without PMA (−PMA) for 16 h. Cell lysates were separated by regular SDS-PAGE (-Phos-tag) or on a 8 % polyacrylamide gel containing 25 mM Phos-tag reagent (+Phos-tag). Upon transfer, membranes were probed with an anti-myc MAb. *Asterisk* marks the slower migrating bands in the Phos-tag gel. **d** Cycling THP-1 cells expressing 3′myc-tagged iso1 were incubated for 14 h with DMSO or one of the CDK inhibitors roscovitine (Rosco), olomoucine II (Olo II), or CDK2-specific inhibitor (CDK2-I) at 0.3 µM or 3.0 µM. After 14 h, cell lysates were separated by SDS-PAGE and analyzed by immunoblot using anti-myc MAb, anti-pSAMHD1-T592 antibody, or anti-HSP90 antibody

The antiviral activity of human SAMHD1 is cell cycle-dependent and is regulated through phosphorylation of the threonine residue at position 592 (T592) by the cellular kinases CDK1 and CDK2, which leads to the loss of antiviral activity in cycling cells [28–31]. Interestingly, both murine SAMHD1 isoforms differ in their C-terminal sequence where the corresponding threonine 603 (T603) is located. While isoform 1 encodes T603 within a CDK1/2 binding site, such a residue is missing in isoform 2 due to differential splicing (Fig. 3a). To determine whether SAMHD1 isoform 1 is phosphorylated at T603, we transiently transfected cycling 293T cells with myc-tagged murine SAMHD1 expression constructs. Two days posttransfection, the cells were lysed and analyzed by SDS-PAGE and immunoblot using an anti-myc antibody and the anti-pSAMHD1-T592 antibody, which specifically recognizes the phosphorylated threonine within the CDK1 binding site (Fig. 3b). We found that, similar to the human protein, mouse SAMHD1 isoform 1, but not the differentially spliced isoform 2 is recognized by the anti-pSAMHD1-T592 antibody in cycling 293T cells. To ensure specificity of the anti-pSAMHD1-T592 antibody, we analyzed SAMHD1 mutants, in which the threonine 592 (human) or 603 (mouse isoform 1) was changed to alanine. While both proteins could be detected with the anti-myc antibody suggesting the proper expression of the mutant proteins, neither protein was recognized by the phospho-specific antibody. Our results indicate that, analogous to the human protein, mouse SAMHD1 isoform 1 is phosphorylated at threonine 603 (T603) in cycling cells. To confirm the phosphorylation of SAMHD1 at T603 we generated human THP-1 cells that lost expression of endogenous human SAMHD1 due to CRISPR/Cas9 endonuclease activity (THP-1 KO). In these cells, we overexpressed muSAMHD1 isoform 1, the phosphorylation-deficient variant T603A and the phosphomimetic mutant T603D, which encodes aspartic acid (D) in the place of threonine at position 603, under the control of a doxycycline (dox)-dependent promotor. The use of a conditional expression systems helps to minimize unwanted effects of SAMHD1 overexpression on THP-1 cell growth and viability. In case of human SAMHD1, the phosphomimetic variant T592D has been shown to functionally replace phosphorylation at T592 and lost its antiviral activity in nondividing cells [28, 31]. To confirm the phosphorylation of muSAMHD1 at T603, we analyzed the cell lysates of proliferating and noncycling, PMA-treated, THP-1 KO cells expressing WT isoform 1, T603A, or T603D by immunoblot. We separated the samples by regular SDS-PAGE or by using Phos-tag acrylamide gels (Wako Chemicals), which allow the detection of a mobility shift of phosphorylated proteins (Fig. 3c) [38, 39]. Western blot analysis revealed multiple bands of WT muSAMHD1 in the Phos-tag gel compared to regular SDS-PAGE. Most strikingly, the slowest migrating band in the Phos-tag gel, which was also absent in regular SDS-PAGE, was clearly visible in dividing cells (−PMA) but strongly reduced in the lysate of noncycling cells (+PMA). Interestingly, the band was also absent in the lysates of cycling T603A-expressing cells suggesting that the phosphorylation in question is T603-specific. Together, these findings indicate that muSAMHD1 is phosphorylated at T603 in cycling cells, thereby supporting our immunoblot analysis with the phospho-specific antibody (Fig. 3b). The muSAMHD1 mutant T603D did not show a similar slow migrating band, suggesting that the aspartate (D) at position 603 is not targeted by the Phos-tag molecules. Human SAMHD1 is phosphorylated at T592 by the cyclin-dependent kinases CDK1 and CDK2 [28, 30, 31]. MuSAMHD1 has also been shown to interact with CDK1 and CDK2 in immunoprecipitation assays [29]. To determine whether the phosphorylation of muSAMHD1 at T603 is also CDK dependent, we preincubated THP-1 KO cells expressing WT isoform 1 with increasing amounts of the CDK inhibitors roscovitine, olomoucine II, and CDK2-specific inhibitor for 16 h prior to cell lysis (Fig. 3d). Probing immunoblots with the phospho-specific antibody revealed that each CDK inhibitor strongly reduced the phosphorylation of muSAMHD1 at T603 at a concentration of 3 µM compared to treatment of the cells with the solvent control DMSO. Incubation with a CDK2-specific inhibitor (Calbiochem) seemed to be very effective and reduced the phosphorylation signal already at a concentration of 0.3 µM and with the least influence on total muSAMHD1 protein level in the lysates (Fig. 3d). These findings additionally confirm the phosphorylation of muSAMHD1 at T603 and suggest that the modification depends on the activity of cyclin-dependent kinases, which is in line with the observed cell cycle-dependence of the T603 phosphorylation (Fig. 3c).

**Fig. 4:**
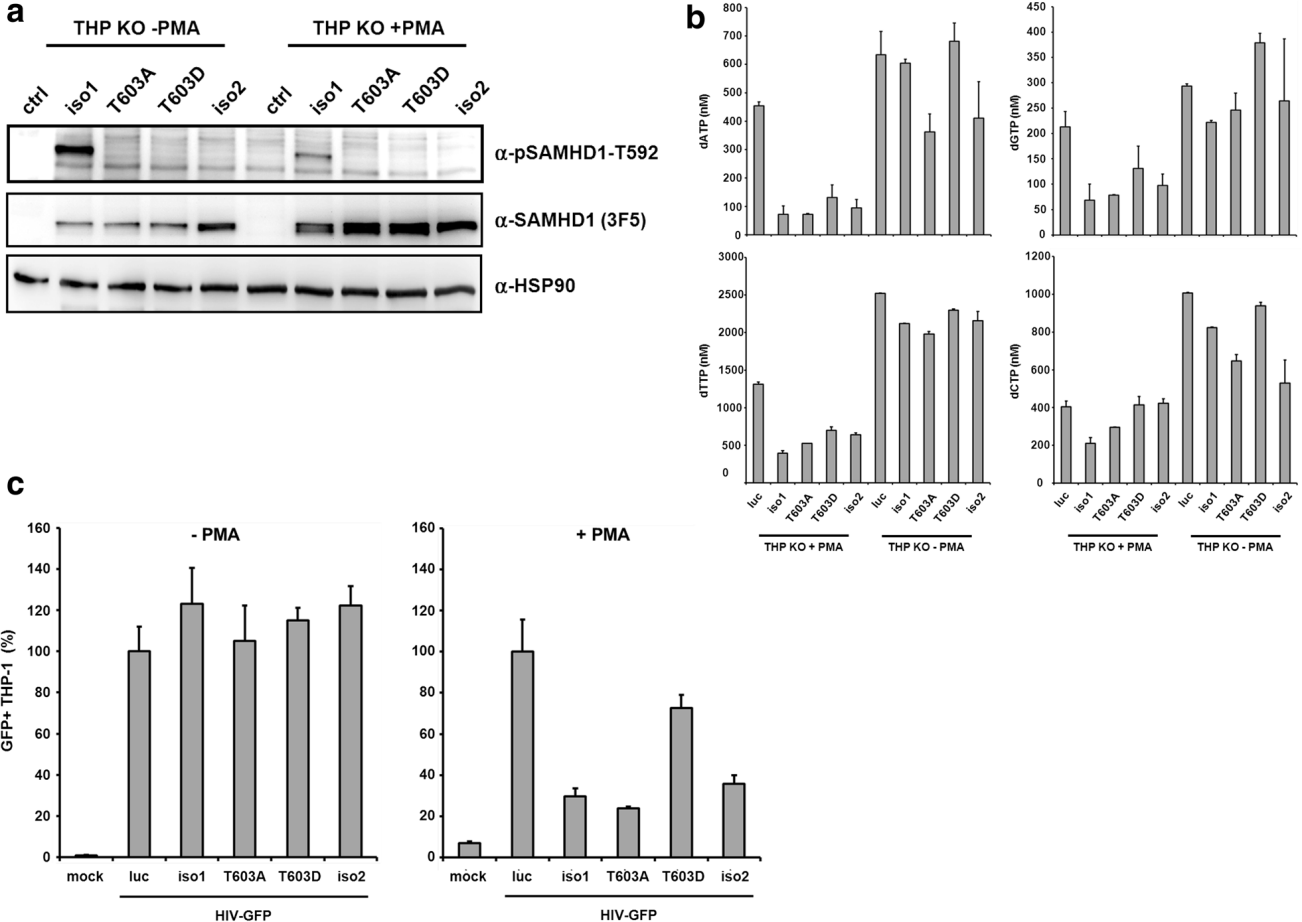
Phosphorylation at threonine 603 regulates the antiviral activity of murine SAMHD1. **a** THP-1 KO cells expressing murine SAMHD1 isoform 1, isoform 1 T603A, isoform 1 T603D, isoform 2, or control cells (luc) were incubated with (+PMA) or without PMA (−PMA) for 16 h, lysed and analyzed by SDS-PAGE and immunoblot. Membranes were probed with anti-HSP90, anti-SAMHD1 MAb 3F5, and anti-pSAMHD1-T592 antibody to determine SAMHD1 phosphorylation. **b** THP-1 KO cells expressing mouse isoform 1, isoform 1 T603A, isoform 1 T603D, isoform 2, or control cells (luc) were differentiated with 50 nM PMA for 24 h or left untreated. After 24 h, samples were normalized to cell number, lysed and dNTP level of 1 × 10^6^ cells were determined using liquid chromatography tandem mass spectrometry. The intracellular concentration of dNTPs (nM) in cycling and noncycling cells is depicted. The dNTP levels are displayed as the average of three measurements with error bars indicating the standard deviation. **c** THP-1 SAMHD1 knockout cells (THP KO) stably expressing mouse iso1, T603A, T603D, iso2 or firefly luciferase expressing control cells (luc) were left untreated (−PMA) or differentiated with 50 nM PMA (+PMA) for 24 h prior to infection with VSV-G/HIV-GFP at a MOI of 1. Three days postinfection, cells were lysed and viral infectivity was quantified by flow cytometry. Viral infectivity was normalized on control cell infection (luc). *Error bars* indicate the standard deviation of triplicate infections. One out of three independent experiments is shown

Next, we asked whether the phosphorylation status of muSAMHD1 correlates with the dNTPase activity and the antiviral restriction of muSAMHD1. First, we analyzed SAMHD1 phosphorylation at T603 in dividing and nondividing THP-1 KO cells expressing muSAMHD1 isoform 1, T603A, T603D, murine isoform 2, and control cells expressing firefly luciferase using a phospho-SAMHD1-specific antibody (Fig. 4a). In cycling cells we only found isoform 1 to be phosphorylated. The T603A mutant and isoform 2, which lacks the CDK binding site, were not detected by the antibody. The phospho-specific antibody did also not recognize the muSAMHD1 variant T603D, indicating that the phosphomimetic aspartic acid (D) cannot replace the phosphate residue within the epitope of the antibody. In PMA-differentiated, noncycling THP-1 cells the signal for phosphorylated muSAMHD1 isoform 1 was strongly reduced indicating that the phosphorylation at T603 is cell cycle-dependent. We then determined the intracellular dNTP level within the different THP-1 KO cells using tandem mass spectrometry (Fig. 4b). In PMA-treated, noncycling cells the expression of muSAMHD1 isoform 1 and isoform 2 strongly reduced the intracellular level of all four dNTPs compared to SAMHD1-negative control cells (luc). In case of dATP, we found that the expression of muSAMHD1 isoform 1 reduced the concentration of the nucleotide by approximately sixfold to 72 nM, which is below the K_m_ of HIV-1 RT for dATP (approximately 100 nM), suggesting suboptimal reverse transcription in these cells [40]. In addition, we also found lower dATP level in cells expressing the variant T603A and the phosphomimetic variant T603D, albeit to a lesser extent. SAMHD1 T603D seemed to reduce dATP level less efficient (130 nM) than T603A (78 nM), which might explain the reduced antiviral activity of this mutant. Similar results were observed for all four dNTPs and are in line with recent findings for human SAMHD1, which also suggest that the phosphorylation at T592 reduces the dNTP hydrolase activity [33–35]. In cycling THP-1 cells, we found the level of dNTPs in all SAMHD1-expressing cells to be enhanced by approximately 7 to 8-fold to levels that would allow efficient HIV reverse transcription. We also observed a slight reduction (1.5-fold) of dATP level in cycling cells expressing SAMHD1 T603A and isoform 2, both of which lack the phosphorylation site at T603, suggesting that these proteins might contain an enhanced dNTPase activity also in cycling cells. However, the intracellular dATP concentrations in these cells (around 400 nM) were still high enough to support efficient HIV-1 reverse transcription [40]. Next, we infected the cycling and noncycling THP-1 KO cells with HIV-GFP reporter virus at a MOI of 1 (Fig. 4c). Three days postinfection, we found no significant difference in HIV-GFP infectivity between the different muSAMHD1-expressing cycling cells (−PMA). However, upon PMA-treatment we found isoform 1, isoform 2, and T603A to inhibit HIV-GFP infectivity by about 3 to 4-fold compared to control cells, whereas the phosphomimetic variant T603D only slightly decreased HIV infectivity. Similar to human SAMHD1, our findings show that the phosphorylation of threonine 603 within the CDK-binding site negatively correlates with the dNTP hydrolase activity and the antiviral activity of murine SAMHD1. Interestingly, isoform 2 and the mutant T603A, which lack the CDK-binding site at T603, were not restricting HIV infectivity in cycling cells.

### SAMHD1 does not inhibit infection in dividing mouse embryonic fibroblasts

**Fig. 5:**
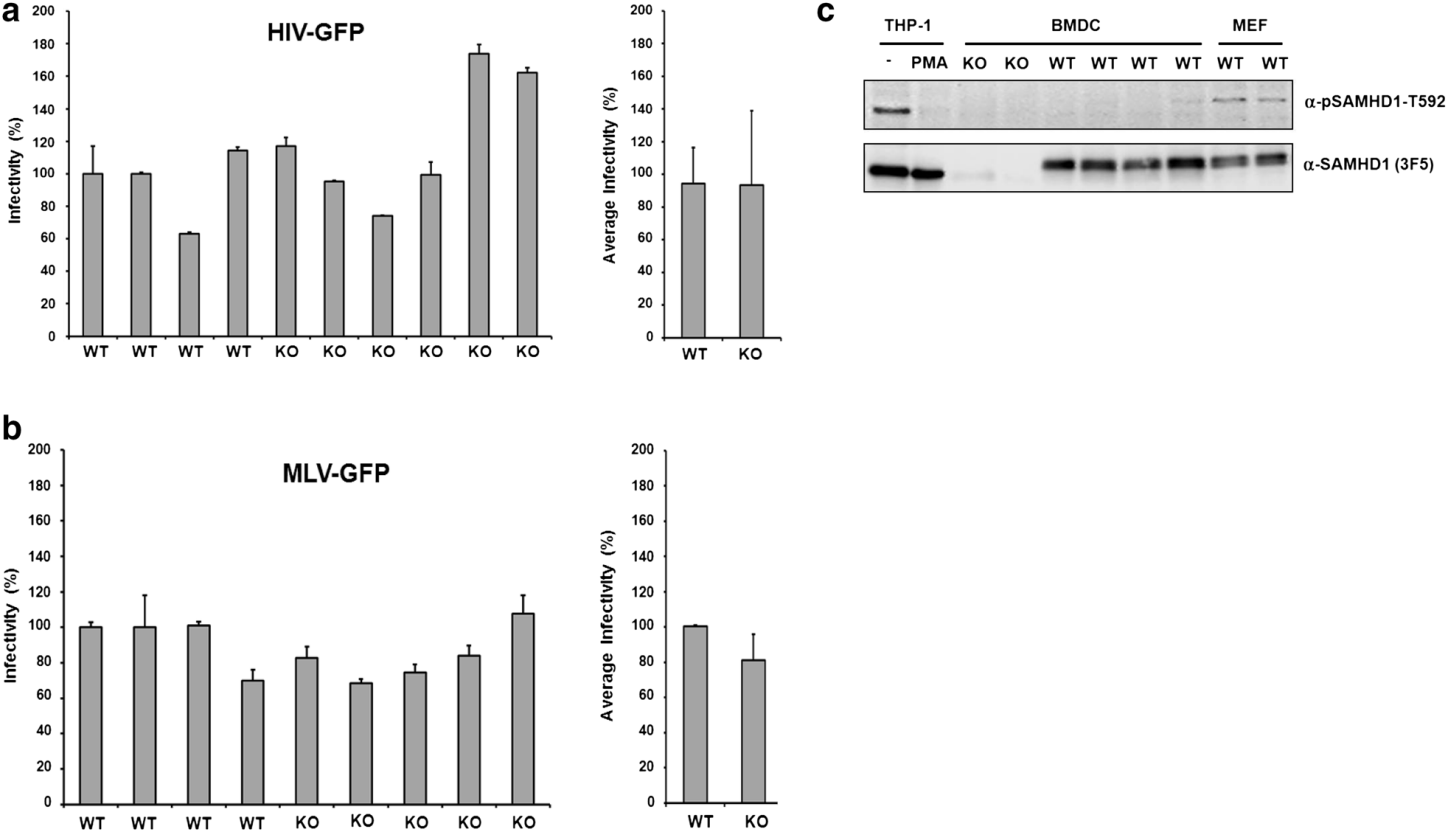
Phosphorylation of SAMHD1 at threonine 603 negatively correlates with infectivity in primary mouse cells. Cycling murine embryonic fibroblasts (MEFs) from SAMHD1 KO and SAMHD1 WT mice were infected with **a** VSV-G/HIV-GFP or **b** VSV-G/MLV-GFP reporter virus at a MOI of 1. Three days postinfection, viral infectivity was quantified by flow cytometry. Viral infectivity is shown as percent infection relative to the infection of cells from a WT mouse. **c** Phosphorylation of murine SAMHD1 at T603 in primary murine cells. MEFs and BMDCs from SAMHD1 KO and WT mice were lysed and analyzed by SDS-PAGE and immunoblot. Lysates from cycling (−PMA) and noncycling human THP-1 cells served as positive and negative control for SAMHD1 phosphorylation. Membranes were incubated with antibodies detecting SAMHD1 (MAb 3F5) and phosphorylated SAMHD1 (anti-pSAMHD1-T592)

Previously, we found that dNTP levels in dividing murine embryonic fibroblasts (MEF) are strongly enhanced in cells from SAMHD1 KO mice compared to WT mice indicating that murine SAMHD1 is an active triphosphohydrolase in primary MEFs [36]. Rehwinkel and colleagues previously showed that both BMDCs and MEFs from SAMHD1 KO mice show enhanced HIV-1 infectivity compared to SAMHD1-positive control cells [37]. However, the authors only found an enhancement of infection when a reporter virus was used that contained an RT mutant that is more sensitive to low dNTP concentrations (V148I) [37]. In contrast, we found that HIV-1 containing WT RT is restricted by SAMHD1 in BMDCs (Fig. 1a) [36]. Thus, we asked whether the replication of WT RT virus is also restricted by SAMHD1 in MEFs. We therefore infected MEF generated from KO and WT mice with HIV-CMVGFP or MLV-EGFP reporter virus at a MOI of 1 and compared the infectivity in these cells by flow cytometry after 72 h. However, in contrast to BMDCs we could not detect significant differences in HIV or MLV reporter virus infectivity between MEF of SAMHD1 KO and WT mice (Fig. 5a, b). We recalculated our previously shown dNTP measurements derived from WT and SAMHD1 KO MEFs to determine the intracellular concentrations of the nucleotides in these cells (data not shown) [36]. Although, we found the dNTP level in SAMHD1-containing MEFs to be 4 to 8-fold lower than in SAMHD1 KO cells, ranging from about 100 nM (dGTP) to 165 nM (dTTP), the dNTP level were still higher than the K_m_ for HIV RT (100 nM), which explains the lack of HIV-1 restriction in MEFs [40]. It is unclear why MLV reporter viruses are not restricted in these cells, since the dNTP level measured are lower than the K_m_ of MLV RT [40]. However, it is conceivable that the subpopulation of dividing cells, which are the target cells of MLV, contain high dNTP levels even in the presence of SAMHD1, especially during S-Phase [41], a fact that might not be reflected by dNTP measurements across the whole MEF population in a sample. Next, we asked whether endogenous SAMHD1 is also phosphorylated in primary mouse cells and analyzed SAMHD1 T603 phosphorylation in primary BMDC and MEFs by western blot. We found murine SAMHD1 to be phosphorylated at T603 in cycling MEFs, where SAMHD1 is antiviral inactive, but not in BMDC, in which SAMHD1 is able to block viral infection (Fig. 5c). Together, our findings show that in primary mouse cells the phosphorylation of endogenous SAMHD1 negatively correlates with its antiviral activity.

### No enhanced degradation of HIV RNA in the presence of SAMHD1

**Fig. 6:**
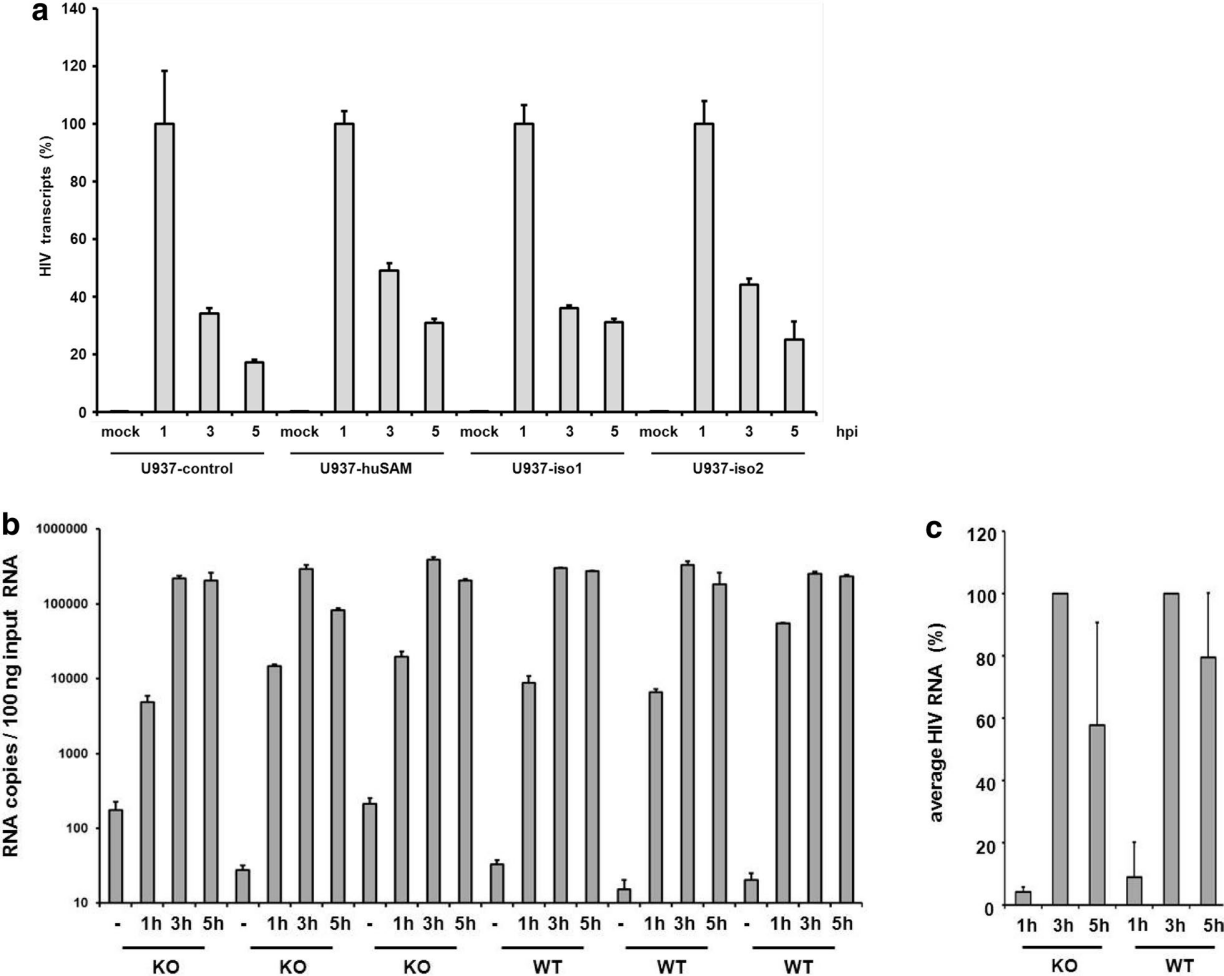
Mouse SAMHD1 does not affect viral RNA level in infected murine cells. **a** PMA-treated U937-control, human SAMHD1 (huSAM), mouse isoform 1 (iso1), and mouse isoform 2 (iso2) expressing cells were incubated with VSV-G/HIV-E2-Crimson reporter virus at a MOI of 1. At several time points postinfection (hours post infection, hpi), the infected cells were lysed and total cellular RNA was isolated, quantified and reverse transcribed. HIV transcripts within the cDNA were quantified by qPCR using oligos targeting the E2-Crimson reporter gene. PCRs on cDNA samples generated in the absence of a reverse transcriptase were used to control for plasmid contamination (not shown). The amounts of HIV transcripts are shown as percent of the HIV RNA content at 1 h postinfection. The average of triplicate infections is shown with *error bars* indicating the standard deviation. One out of three independent experiments is shown. **b** BMDC from SAMHD1 KO (n = 3) or WT (n = 3) mice were infected with VSV-G/HIV-E2-Crimson reporter virus at a MOI of 1. Similar to (**a**), viral RNA from uninfected cells (−) or infected cells was isolated at various time points postinfection, quantified and is depicted as number of HIV RNA copies/100 ng input RNA. The *columns* represent the average of triplicate infections with *error bars* indicating the standard deviation. One of two independent experiments is shown. **c** The average amount of viral RNA copies in infected cells from WT (n = 3) or KO mice (n = 3) at different time points postinfection as shown in (**b**). HIV transcripts were quantified relative to the maximum amount of transcripts at 3 h postinfection

It has been shown that SAMHD1 contains an additional nuclease activity and it has been suggested that the direct degradation of incoming HIV RNA is responsible for the inhibition of infection [27]. Thus, we asked whether the recently proposed RNase activity of human SAMHD1 might contribute to this reduced infectivity in the mouse. We therefore infected human and mouse SAMHD1-expressing U937 cells and primary BMDC with HIV reporter virus and compared the viral RNA content of SAMHD1-positive and negative cells at several time points postinfection (Fig. 6). In PMA-differentiated U937 cells, we found high levels of HIV RNA in the cells after 1 h and a strong decrease of viral RNA content 3 and 5 h postinfection (Fig. 6a). However, we could not detect any differences in the rate of degradation of viral RNA after 3 h and 5 h between SAMHD1 expressing or control cells (Fig. 6a). Our finding indicates that human or mouse SAMHD1 does not influence viral RNA stability in infected U937 cells. In primary murine BMDC, we found the peak viral RNA load to be reached later postinfection and the decay of viral RNA to be slower than in the U937 cells (Fig. 6b). However, comparing the amount of viral RNA in WT and KO BMDC over time, muSAMHD1 did not have a significant impact on total viral RNA content at different time points postinfection (Fig. 6c). These results suggest that similar to the human protein, exogenous and endogenous mouse SAMHD1 does not influence viral RNA stability in newly infected cells.

## Conclusion

A range of diverse retroviruses, retroelements, and even DNA viruses are restricted by the host restriction factor SAMHD1. To better understand the influence of SAMHD1 on the replication of these viruses, a small animal model could be key. In this study, we characterize the antiviral activity of endogenous mouse SAMHD1 using our previously described SAMHD1 KO mouse model.

By comparing BMDC from WT and SAMHD1 KO mice we found that murine SAMHD1 blocks HIV-1 and MLV reporter virus infection in primary cells at the level of reverse transcription. Previously, we and others detected increased cellular dNTP levels in various cell types of SAMHD1 KO mice, including BMDC [36, 37]. Correlating the block to reverse transcription and the reduced dNTP levels in SAMHD1-expressing primary cells suggests that the observed dNTP hydrolase activity of SAMHD1 is an important factor for virus restriction in the mouse.

The antiviral activity of human SAMHD1 is regulated through phosphorylation at threonine 592 within a CDK1 binding site, making the posttranslational phosphorylation of muSAMHD1 a good candidate for this additional regulation. Similar to the human protein, we found that muSAMHD1 isoform 1 is phosphorylated at the corresponding threonine 603 (T603) in cycling cells. We used a previously described SAMHD1 phosphoT592-specific antibody and Phos-tag polyacrylamide gels to confirm the phosphorylation of muSAMHD1 at T603. We found that the antiviral activity of muSAMHD1 correlates with the unphosphorylated threonine at position 603 in nondividing THP-1 cells. In cycling cells, we found that the phosphorylation of SAMHD1 at T603 within the CDK1-binding site can indeed be blocked by various CDK inhibitors, which additionally confirms the cell cycle-dependence of the observed phosphorylation. Of note, being aware that most CDK inhibitors are blocking multiple CDKs with varying efficiencies, we never aimed at isolating a certain kinase that dominantly phosphorylates muSAMHD1. However, giving the fact the CDK2 inhibitor shows a very prominent effect in our assays and that it has been shown previously that muSAMHD1 immunoprecipitates CDK2, and to a lesser extent CDK1, it is tempting to speculate that CDK2 and CDK1 are primarily responsible for muSAMHD1 phosphorylation at T603 [29].

In differentiated THP-1 cells the phosphorylation-deficient mutant T603A blocked viral infection, while the phosphomimetic variant T603D was inactive. Using a SAMHD1 phosphoT592-specific antibody, we found that the phosphorylation at T603 also correlates with the antiviral activity in primary mouse cells. Our finding that SAMHD1 is phosphorylated in permissive, cycling MEFs but unphosphorylated in restrictive, nondividing BMDCs suggests that, similar to human SAMHD1, the antiviral activity of SAMHD1 in the mouse is also regulated in a cell cycle-dependent manner. In line with this observation, we also found that the T603A-mutant of SAMHD1, which lacks the regulatory CDK-binding site, is not antiviral active in cycling THP-1 cells. This finding suggests that, in addition to the regulatory phosphorylation at T603, the phase of the cell cycle might also play an important role in regulating the antiviral activity of muSAMHD1. Similarly, muSAMHD1 isoform 2 should be constantly active and display an enhanced antiviral activity due to its lack of the inactivating phosphorylation site. However, we found that isoform 2 is only antiviral active in noncycling THP-1 cells and inactive in cycling cells, further confirming the cell cycle-dependence of the block. Little is known about the activity and abundance of SAMHD1 isoform 2 in vivo, which is mainly due to the fact that no isoform 2-specific antibody is available to date. Our finding that in primary myeloid cells and fibroblasts the phosphorylation of muSAMHD1 at T603 strongly correlates with its antiviral activity suggests a rather minor role for isoform 2 in antiviral restriction in these cell types. However, it is conceivably that isoform 2 plays an essential role in antiviral defense in a different cell type or against a different threat, like DNA viruses or endogenous retroviruses and retroelements.

Similar to human SAMHD1, we found that the phosphomimetic muSAMHD1 mutant T603D is enzymatically less active and depletes dNTPs in PMA-differentiated THP-1 cells not as efficient as the WT protein. The relatively small difference in activity results in intracellular dNTP level above the threshold needed for efficient RT and therefore abrogates the block to viral infection (Fig. 4). Recently, an additional RNase activity of human SAMHD1 has been described as alternative mechanism of restriction [27]. Therefore, we tested whether enhanced viral RNA degradation in the presence of muSAMHD1 could account for a dNTP-independent activity. To analyze the role of RNA degradation in antiviral restriction, we compared HIV RNA levels in human and murine SAMHD1-expressing U937 cells and primary BMDC. However, in contrast to the described phenotype, we did not find enhanced RNA degradation in the presence of human or murine SAMHD1 in these cells. Thus, the mechanism of SAMHD1-mediated restriction in the mouse rather depends on the block to reverse transcription than on the degradation of viral genomic RNA. Why we did not detect an HIV RNA degrading activity for human SAMHD1 in our assays is not clear. However, the potential effect of human SAMHD1 on RNA seems to be rather complicated since several other groups also could not detect a RNase activity of human SAMHD1 [21, 24, 26, 42].

Previously, we showed that SAMHD1 does not influence the in vivo replication of Friend MLV, a murine retrovirus. We therefore analyzed the activity of mouse SAMHD1 against a MLV reporter virus and found that muSAMHD1 also blocks the reverse transcription of MLV in BMDC and murine SAMHD1-expressing U937 cells. Thus, murine SAMHD1 is in principal able to restrict MLV reporter virus, but does not affect Friend virus replication in vivo. The most likely explanation for this finding is that SAMHD1 is not active in proliferating erythroblast precursors, the main target cells of FV. SAMHD1 is thought to be active in nondividing cells only, which are not infected efficiently by murine leukemia viruses due to their inability to transfer the viral cDNA into the nucleus. Replication in cell types where SAMHD1 is inactive could therefore be an efficient strategy for a virus to bypass the SAMHD1-mediated block. The fact that mouse SAMHD1 is only antiviral active in cells that are typically not infected by murine leukemia virus suggests that exogenous retroviruses might not be the prime target of SAMHD1 restriction in the mouse. It has been described that the replication of various DNA viruses as well as retroelements are blocked by human SAMHD1 [15, 16, 43]. Therefore, it will be very interesting to analyze the replication of murine DNA viruses, endogenous retroviruses and retroelements in the SAMHD1 KO mouse model.

Together, our data suggest that the dNTP hydrolase activity of muSAMHD1 is very important for the antiviral restriction in the mouse. In addition, we found that, similar to human SAMHD1, the antiviral activity of muSAMHD1 is regulated through a cell cycle-dependent phosphorylation at T603 and that this phosphorylation also correlates with the antiviral activity of muSAMHD1 in primary mouse cells. Our results also show that the antiviral restriction mediated by murine SAMHD1 is mechanistically similar to what is known for the human protein. This finding proves SAMHD1 KO mice to be a very valuable tool to analyze the replication of different viruses, including retroviruses, retroelements, and DNA viruses, with regard to SAMHD1 restriction in vivo.

## Methods

### Cells and cell culture

293T cells were cultured in Dulbecco’s modified Eagle medium/10 % fetal bovine serum (FBS). U937 and THP-1 cells were cultured in RPMI 1640/10 % FBS. Monocytic U937 and THP-1 cells were differentiated by culturing for 1 day with 50 nM phorbol 12-myristate 13-acetate (PMA). U937 cells stably overexpressing murine SAMHD1 isoform 1 or 2 were generated by lentiviral transduction. Three days postinfection with lentiviral particles, cells were selected and cultivated in the presence of 100 µg/ml geneticin (Invitrogen). THP-1 knockout (KO) SAMHD1 cells were generated using the CRISPR/Cas9 endonuclease system. THP-1 KO SAMHD1 cells expressing the murine SAMHD1 isoform 1, isoform 2, or the isoform 1 mutants T603A and T603D were generated by transducing THP-1 KO SAMHD1 cells with lentiviral particles containing SAMHD1 expression vectors based on pLVX-Tight-Puro (Clontech), which express the different SAMHD1 proteins under the control of doxycycline, and lentiviral particles based on pLVX tet-on advanced, which expresses the reverse Tet transactivator. The transduced cells were cultivated in the presence of 100 µg/ml geneticin (G418) and 2.5 µg/ml puromycin. To induce muSAMHD1 expression, cells were treated with 500 ng/ml doxycycline for 72 h. To generate bone marrow derived dendritic cells (BMDC), bone marrow was prepared from tibia and femur of SAMHD1-deficient and control mice as described previously [36]. Briefly, bone marrow cells were differentiated in vitro for 7 days in RPMI 1640 Medium (Biochrom) supplemented with 10 % heat-inactivated fetal calf serum (Sigma), 10 ng/ml murine GM-CSF (PeproTech), 100 U/ml Penicillin, 100 µg/ml Streptomycin (Biochrom), and 2 mM L-Alanyl l-glutamine (Biochrom).

### Generation of THP-1 CRISPR/Cas9 KO cell lines

THP-1 cells were plated at a density of 2 × 10^5^ cells/ml. After 24 h, 2.5 × 10^6^ cells were resuspended in 250 µl Opti-MEM, mixed with 5 µg CRISPR/Cas plasmid DNA, and electroporated in a 4-mm cuvette using an exponential pulse at 250 V and 950 mF utilizing a Gene Pulser electroporation device (Bio-Rad Laboratories). We used a plasmid encoding a CMV-mCherry-Cas9 expression cassette and a human SAMHD1 gene specific gRNA driven by the U6 promoter [44]. An early coding exon of the SAMHD1 gene was targeted using the following gRNA construct: 5′-CGGAAGGGGTGTTTGAGGGG-3′. Cells were allowed to recover for 2 days in 6-well plates filled with 4 ml medium per well before being FACS sorted for mCherry-expression on a BD FACS Aria III (BD Biosciences). For subsequent limiting dilution cloning, cells were plated at a density of 5, 10, or 20 cells per well of nine round-bottom 96-well plates and grown for 2 weeks. Plates were scanned for absorption at 600 nm and growing clones were identified using custom software and picked and duplicated by a Biomek FXp (Beckman Coulter) liquid handling system. One duplicate was used to recover gDNA and characterize the gene editing as previously described [45].

### Plasmids

For exogenous expression of myc-tagged muSAMHD1 proteins in U937 cells, the isoform 1, isoform 2, muSAMHD1 T603A, and muSAMHD1 T603D were cloned into the lentiviral vector p6NST56myc-IRES-EYFP. Mutations of threonine 603 were introduced into codon-optimized muSAMHD1 by overlapping PCR mutagenesis. The amplicons were digested with *Xba*I and *Not*I and cloned into p6NST56myc-IRES-EYFP. The resulting constructs were confirmed by nucleotide sequencing. For expression of codon-optimized myc-tagged muSAMHD1 proteins in THP-1 KO cells under the control of doxycycline, isoform 1, isoform 2, and the mutant proteins T603A and T603D were amplified by PCR and cloned into the lentiviral vector pLVX-Tight-Puro via the restriction sites for the endonucleases *Xba*I and *Not*I. The resulting constructs were confirmed by nucleotide sequencing. The plasmids used to generate SIV VLPs, pSIV3+ and pSIV3+ X−, have been described previously and are based on SIVmac251 [46]. The env-deficient HIV-1 reporter plasmids pNL43-E^—^CMVGFP and pNL-luc3-E and the MLV reporter plasmid pMXSfi-EGFP have been described previously [12, 47]. The plasmid pNL43-E^—^CMVGFP encodes GFP under the control of an internal CMV promoter, while the expression of luciferase in pNL-luc3-E^−^ is driven by the HIV-1 LTR promotor. The *env*-deficient MLV reporter plasmids SF91.EGFP (MLV + PBS) and pSF91ΔPBS.EGFP have been described previously [48–50].

### Virus preparation and infections

Reporter virus was produced in 293T cells by co-transfecting *env*-deficient reporter virus plasmids and a vesicular stomatitis virus glycoprotein expression plasmid (pVSV-G) using the calcium phosphate precipitation method. HIV-1-CMVGFP (NL43-CMVGFP) was generated by co-transfection of pNL43-E^−^-CMVGFP and pVSV-G at a mass ratio of 4:1. NL4-3-E2-Crimson was generated by co-transfection of pCSE2W E2-Crimson-reporter construct and pVSV-G at a mass ratio of 4:1. Murine leukemia reporter virus (MLV-EGFP) was generated by co-transfecting pMXSfi-EGFP and pVSV-G into 293-GAGPOL packaging cells, which stably express MLV gagpol. To analyze MLV reverse transcription by qPCR, MLV + PBS and MLV-PBS reporter viruses were generated by co-transfection of pSF91.EGFP (MLV + PBS) or pSF91ΔPBS.EGFP (MLV-PBS) and pVSV-G at a mass ratio of 4:1 in 293-GAGPOL packaging cells. To generate SAMHD1-expressing cell lines, lentiviral particles encoding codon-optimized murine WT SAMHD1, SAMHD1 T603A, SAMHD1 T603D, or control particles were generated by co-transfection of p6NST56myc-mucoSAMHD1-IRES-EYFP or pLVX-Tight-Puro-myc-mucoSAMHD1, pCMVdeltaR8.91, and pVSV-G. To produce VLPs, 293T cells were co-transfected with pSIV3 + , or pSIV3 + X- and pVSV-G at a mass ratio of 2:1. For all transfections the culture medium was replaced after 6 h. Supernatants were harvested 48 h posttransfection, passed through 0.4 μm pore size filters and purified by pelleting through a 20 % sucrose cushion at 32,000 g for 2 h at 4 °C. The pellets were resuspended in RPMI 1640, aliquoted and frozen at −80 °C. All GFP- or E2-Crimson- expressing reporter viruses were titered on 293T cells and infectivity was determined 72 h postinfection by flow cytometry. In reporter virus infection assays BMDC, THP-1, or U937 cells (3.0 × 10^5^) were spin-infected in 1 ml RPMI for 2 h at 500 × g with GFP or E2-Crimson reporter virus at a MOI of 1. The number of infected cells was determined 3 days later by flow cytometry.

### Quantitative PCR

BMDC or PMA-treated U937 were infected at a MOI of 1 with HIV or MLV reporter virus stocks pretreated for 1 h with 50 U/ml Benzonase (Sigma) to remove contaminating plasmid DNA. 20 µM Nevirapine was added to one sample 14 h prior to infection to control for residual HIV-1 plasmid DNA. To control for contaminating MLV plasmid DNA, reverse transcription-incompetent MLV reporter virus lacking the primer binding site (MLV-PBS) was included in the experiments. After 12 and 24 h, total cellular DNA was isolated using a QIAamp DNA Mini Blood Kit (Qiagen). Reverse transcripts in 65 ng of total DNA were quantitated by qPCR on an ABI Prism 7300 cycler (Applied Biosystems) using SYBR green reagent (Thermo Scientific). The oligonucleotides used to quantify HIV-1 late RT transcripts were, 5′-HIV-lateRT (TGTGTGCCCGTCTGTTGTGT) and 3′-HIV-lateRT (GAGTCCTGCGTCGAGAGAGC). The primer pairs used to detect MLV-GFP reverse transcripts were GFP-qPCR-for (CCTGAAGTTCATCTGCACCA) and GFP-qPCR-rev (GGTCTTGTAGTTGCCGTCG). Standard curves were generated using serially diluted proviral plasmids.

### Quantitative RT-PCR

Total RNA of U937 cells or BMDC infected with VSV-G/HIV-CMVGFP reporter virus (MOI of 1) was isolated at 1 h, 3 h, and 5 h posttransduction using the NucleoSpin RNA Kit (Machery-Nagel) according to the supplier’s manual. One microgram of purified, DNase I-treated RNA was reverse transcribed using Oligo-dT primer. Quantitative PCR was performed in triplicates on an ABI Prism 7300 cycler (Applied Biosystems) using 330 ng cDNA, the reporter gene-specific primers GFP-qPCR-for (5-CCTGAAGTTCATCTGCACCA-3), GFP-qPCR-rev (5-GGTCTTGTAGTTGCCGTCG-3) for BMDC infection or 5′ qPCR Crimson (5-GCGCTTCAAGGTGCACATGG-3) and 3′ qPCR Crimson (5-GGTGCCCTCGTAGGGCTTGC-3) for U937 infections, and SYBR green reagent (Thermo Scientific). The data were normalized to amplified transcripts of the internal control gene GAPDH using the oligos GAPDH-For (5-AAGGGGCGGAGATGATGAC-3) and GAPDH-Rev (5- GGTGCTGAGTATGTCGTGGAG-3).

### Immunoblot analysis

Cells were lysed in NP40 lysis buffer (10 mM TrisHCl, 150 mM NaCl, 2 mM EDTA, 0.5 % NP-40, Halt Protease Inhibitor). Lysates were quantified by Bradford assay (Carl Roth). In general, 30 μg per sample were separated by SDS-PAGE, transferred to PDVF membranes and probed with mouse anti-SAMHD1 MAb 3F5 (Origene), rabbit anti-pSAMHD1 T592 (ProSci), or mouse anti-HSP90 MAb (Santa Cruz). The membranes were then probed with anti-mouse or anti-rabbit horseradish peroxidase (HRP)-labeled secondary antibodies (GE-Healthcare) and visualized using HRP substrate (Pierce) on an Intas Advanced Fluorescence Imager (Intas). For CDK inhibitor studies, cycling THP-1 KO cells expressing isoform 1 of muSAMHD1 were incubated with DMSO or one of the CDK inhibitors roscovitine, olomoucine II, or CDK2-specific inhibitor (Calbiochem) at 0.3 µM or 3.0 µM for 14 h prior to cell lysis. For Phos-tag analysis, cells were lysed in EDTA-free NP40 lysis buffer. Samples were separated on 8 % polyacrylamide gels containing 25 µM Phos-tag Reagent (Wako Pure Chemicals) and 100 µM MnCl_2_. Upon washing the gels with transfer buffer containing additional 10 mM EDTA, proteins were transferred onto PDVF membranes. Membranes were probed with mouse anti-myc (Santa-Cruz) and anti-mouse horseradish peroxidase (HRP)-labeled secondary antibodies (GE-Healthcare).

### Quantitation of endogenous nucleoside triphosphates

Intracellular dNTP concentrations were determined by liquid chromatography-tandem mass spectrometry as described previously [51]. Briefly, 1 × 10^6^ THP-1 cells were pelleted, lysed and analytes were extracted by protein precipitation with methanol after spiking samples with stable isotopically labeled analogues of the dNTP (used as internal standards). Samples were chromatographically separated using an anion exchange HPLC column and analyzed in a 5500 QTrap mass spectrometer, operated as triple quadrupole in positive multiple reaction monitoring (MRM) mode. The calibration ranges were 0.5–500 ng/mL in the injected solution for dATP and dGTP and 1–1000 ng/mL for dCTP and dTTP, respectively.
